# Role of Fetoscopic Airway Evaluation Immediately Preceding EXIT Procedure in Fetuses With Suspected Airway Obstruction

**DOI:** 10.1002/pd.70243

**Published:** 2026-09-11

**Authors:** Daniel Sanin‐Ramirez, Joan Sabria, Josep M. Martinez, Mar Bennasar, Montserrat Izquierdo, Elisenda Eixarch, Eduard Gratacos

**Affiliations:** ^1^ BCNatal Fetal Medicine Research Center (Hospital Clínic and Hospital Sant Joan de Déu) Universitat de Barcelona Barcelona Spain; ^2^ Institut d’Investigacions Biomèdiques August Pi i Sunyer (IDIBAPS) Barcelona Spain; ^3^ Facultat de Medicina i Ciències de la Salut Universitat de Barcelona (UB) Barcelona Spain; ^4^ Institut de Recerca Sant Joan de Déu (IRSJD) Barcelona Spain; ^5^ Institut d’Investigacions Biomèdiques August Pi i Sunyer (IDIBAPS) and Centre for Biomedical Research on Rare Diseases (CIBERER) Barcelona Spain; ^6^ Neonatal Unit Hospital Sant Joan de Déu Universitat de Barcelona Barcelona Spain

**Keywords:** airway obstruction, congenital abnormalities, Ex Utero Intrapartum Treatment (EXIT), fetal surgery, fetoscopy

## Abstract

**Objective:**

This study describes the role of pre‐EXIT (Ex Utero Intrapartum Treatment) fetoscopic airway evaluation in fetuses with suspected airway obstruction.

**Methods:**

Single center, retrospective, observational study including fetuses with suspected airway obstruction undergoing fetoscopic airway evaluation prior to EXIT between 2013 and 2025.

**Results:**

Nine fetuses underwent EXIT with previous fetoscopy at a median gestational age of 35 + 6 weeks (IQR 34 + 4, 37 + 1). The most frequent indications were cervical teratoma (*n* = 3) and lymphangioma (*n* = 3). Fetoscopy confirmed airway patency in one case (11.1%), allowing de‐escalation to conventional cesarean section. Airway was secured by endotracheal intubation in seven cases (77.8%) and tracheostomy was required in one (11.1%). Survival at the delivery room was 100%, and one neonate died prior to discharge.

**Conclusion:**

Fetoscopic airway evaluation prior to EXIT is a valuable adjunct in fetuses with suspected airway obstruction. It can confirm the need for EXIT, optimize the trajectory for airway access, and potentially avoid tracheostomy, improving the safety and efficiency of airway management.

## Summary

- What's already known about this topic?
  ◦ Prenatal assessment of potential fetal airway obstruction is key for delivery planning; EXIT is often used to secure the fetal–neonatal airway.
  ◦ Fetoscopic airway evaluation can provide laryngoscopy‐like visualization and may be diagnostic and occasionally therapeutic.
- What does this study add?
  ◦ Pre‐EXIT fetoscopy is feasible and can change management: confirming patency, allowing EXIT avoidance, mapping or guiding the intubation route and potentially preventing tracheostomy.

## 1 Introduction

Congenital cervical and oropharyngeal tumors, intrinsic airway anomalies, or severe craniofacial malformations can severely distort fetal airway anatomy, creating a significant risk of obstruction at birth. Securing a safe airway at delivery may be extremely challenging, potentially requiring emergent tracheostomy with a high risk of hypoxia, brain injury, and death [1, 2]. Early prenatal identification of fetuses at risk is therefore crucial for planning delivery, often requiring the Ex Utero Intrapartum Treatment (EXIT) procedure to ensure a controlled establishment of the fetal‐neonatal airway [3]. First described in 1993 and refined in 1997 [4, 5], EXIT was initially applied in congenital diaphragmatic hernia (CDH) to reverse tracheal occlusion, but its indications have since expanded to a range of intrinsic and extrinsic causes of airway obstruction [2, 6, 7, 8, 9]. The technique requires adequate uterine relaxation and fetal anesthesia/immobilization, most commonly achieved with maternal general anesthesia. However, regional anesthesia combined with adjunctive tocolytic agents has also been successfully used in selected cases to maintain the degree of uterine relaxation required for the procedure. This enables partial delivery and surgical access to the fetus while preserving uteroplacental circulation and gas exchange for up to 40–60 minutes [3, 10]. Continuous monitoring is essential due to maternal risks of hemorrhage and uterine atony. Airway management can be performed by initial laryngoscopy, followed if necessary by bronchoscopy, tracheostomy, or mass resection; in unsuccessful cases, transfer from EXIT to Extracorporeal Membrane Oxygenation (ECMO) has been described [11].

Predicting the degree and type of airway obstruction remains a major challenge when planning an EXIT. While ultrasound and MRI provide essential information, they cannot reliably anticipate the feasibility or difficulty of intubation. The oropharynx and upper airways may be severely distorted due to mass compression, rendering conventional intubation very challenging and prompting surgeons to resort to tracheostomy. Experience with fetal endoscopic tracheal occlusion in CDH has demonstrated the feasibility and safety of endoscopic access with direct visualization of the larynx and trachea [12, 13]. Extending this rationale to EXIT, fetoscopic airway evaluation has been proposed as an adjunct that can replicate neonatal laryngoscopy, but in optimal conditions provided by a fluid environment and without the time pressure of an examination under EXIT. Beyond diagnostic clarification and confirmation of the need for EXIT, fetoscopy may help mapping an intubation trajectory or enable placement of guiding catheters, reducing the time to intubation and the need for more invasive steps such as tracheostomy.

This study describes the role of pre‐EXIT fetoscopy in a series of cases with suspected airway obstruction, specifically its capacity to confirm the need for EXIT, inform in the strategy for securing airway access, and avoid unnecessary more invasive procedures such as tracheostomy when the airway is found to be patent.

## 2 Materials and Methods

Retrospective, observational study at one tertiary‐national referral center for fetal surgery and maternal‐fetal medicine, including all fetuses with suspected airway obstruction who underwent fetoscopic airway evaluation prior to the EXIT procedure. Medical records were reviewed for all consecutive cases managed between 2013 and 2025, beginning from the year when we started systematic use of pre‐EXIT fetoscopy. The study was approved by the Ethics Committee (code HCB/2025/1083). Inclusion criteria were pregnancies with suspicion of airway obstruction based on prenatal imaging, availability of complete image studies (ultrasound and/or fetal MRI), detailed documentation of the fetoscopic and EXIT procedures, and neonatal outcomes. Exclusion criteria were cases where fetoscopy was not performed prior to EXIT.

### 2.1 Prenatal Assessment

All patients underwent detailed fetal evaluation including morphologic two‐ and three‐dimensional ultrasound and fetal MRI when indicated, contributing to the identification of the level and extent of the obstruction as well as characterizing the type and size of the cervical mass. The suspicion of airway compromise was based on findings such as severe tracheal displacement, absence of visible tracheal lumen, polyhydramnios, and mass effect on the upper aerodigestive tract. In all cases, a multidisciplinary team (fetal medicine specialists, obstetricians, anesthesiologists, pediatric surgeons, neonatologists, and otolaryngologists if needed) eventually reviewed the case and determined the need for fetoscopic evaluation or EXIT planning.

### 2.2 Fetoscopic Procedure

Fetoscopic airway evaluation was scheduled electively immediately prior to EXIT delivery. The procedure was performed under maternal peridural and/or general anesthesia depending on maternal conditions. Fetal intramuscular anesthesia was administered under ultrasound guidance to the fetal thigh, using the following drug regimen: fentanyl at a dose of 10–15 μg/kg, vecuronium at 0.1–0.2 mg/kg, and atropine at 20 μg/kg. After locating a placenta‐free uterine entry site and positioning the fetal face, a percutaneous 10 Fr trocar was inserted into the amniotic sac under real‐time ultrasonographic and color Doppler guidance. A 3.3‐mm semi‐rigid fetoscope (Karl Storz 11,540, Tuttlingen, Germany) was introduced into the amniotic sac through the 10 Fr cannula, and then into the fetal oral cavity. The scope was advanced to visualize the larynx, vocal cords, upper trachea and carina. Airway patency and the degree of intubation difficulty were assessed by direct visualization. In cases where patency was confirmed, the procedure was terminated, and immediate cesarean section or vaginal delivery induction was performed. If airway obstruction, such as tracheal collapse, was confirmed during fetoscopic evaluation, an EXIT procedure was performed to secure the airway.

## 3 EXIT Procedure

Uterine relaxation was maintained using inhaled anesthetic agents titrated to achieve adequate tone during the procedure. A low transverse (Pfannenstiel) skin incision was performed, followed by a layered dissection to access the peritoneal cavity. The uterus was exposed, and a low transverse uterine incision (Kerr‐type) was made avoiding the placenta; a hemostatic stapling device was used to secure the edges of the hysterotomy. Continuous infusion of warm isotonic saline was used to minimize fluid loss and maintain intrauterine volume, as well as fetal temperature. Partial delivery of the fetus was then performed, exposing only the fetal head, neck, and at least one arm while maintaining the fetal torso inside the uterine cavity. Airway management was individualized based on the anatomical features of the obstruction. Options included direct laryngoscopy, rigid bronchoscopy, or tracheostomy. After successful airway establishment and confirmation of effective ventilation, the umbilical cord was clamped, the infant was fully delivered, and uterine tocolytics were discontinued to facilitate uterine contraction and minimize maternal hemorrhage.

### 3.1 Postnatal Management

All neonates were received in the operating room by a neonatal team. Airway patency and endotracheal tube position were confirmed clinically and radiographically. Neonates were admitted to the neonatal intensive care unit (NICU) for ongoing monitoring and support. Definitive management of the cervical mass was performed in different time lapses based on the diagnosis, postnatal imaging and airway stability.

### 3.2 Statistical Analysis

Statistical analyses were performed using Stata version 18. Categorical variables are reported as counts and percentages. Continuous variables are presented as mean ± standard deviation for normally distributed data, or as median (Q1, Q3) for non‐normally distributed data. Normality was assessed using the Shapiro–Wilk test; a *p*‐value ≤ 0.05 was considered indicative of non‐normal distribution. Statistical significance was defined as a *p*‐value < 0.05.

## 4 Results

During the study period, nine cases underwent EXIT therapy following prior fetoscopy; their clinical characteristics are summarized in Table 1. Fetal anomalies were diagnosed at a median gestational age of 27 + 4 weeks (IQR 25+0–30 + 6 weeks). Polyhydramnios was observed in 66.7% of cases, and amniodrainage was required in four patients. Premature uterine contractions occurred in three patients, requiring tocolytic therapy; in two of them, progression of symptoms made it necessary to advance the procedure, while in one patient, preterm premature rupture of membranes likewise necessitated an earlier intervention. Prenatal diagnoses were cervical teratoma (3) or lymphangioma (3), bronchogenic cyst extending from the mediastinal into the cervical region, resulting in significant displacement of the tracheoesophageal complex (1), congenital high airway obstruction syndrome (CHAOS) due to laryngeal atresia, and Pierre Robin sequence with severe micrognathia (1). All patients had normal genetic evaluation by chromosomal microarray analysis. Additionally, the fetus diagnosed with the Pierre Robin sequence underwent whole exome sequencing with normal findings.

**TABLE 1:** Maternal and fetal characteristics in cases managed with fetoscopy and EXIT.

	*n* = 9
Age (years)	35.4 (32.7, 35.8)
Parity	
Parous	6 (66.7%)
Nulliparous	3 (33.3%)
BMI (kg/m^2^)	28.9 (23.0, 33.0)
Gestational age at diagnosis (weeks + days)	27^+4^ (25^+0^, 30^+6^)
Diagnosis	
Teratoma	3 (33.3%)
Lymphangioma	3 (33.3%)
Cervical bronchogenic cyst	1 (11.1%)
Laryngeal atresia (CHAOS)	1 (11.1%)
Pierre robin sequence	1 (11.1%)
Placenta location	
Posterior	5 (55.6%)
Anterior	4 (44.4%)
Polyhydramnios *n* = 6 (66.7%)	
Amniotic fluid index (cm)	35.5 (30.0, 43.0)
Amniodrainage	4 (44.4%)
PPROM	1 (11.1%)
Threaten preterm delivery	3 (33.3%)

*Note:* Categorical variables are described as number (%), and numerical variables as median (Q1, Q3).

Abbreviations: BMI, body mass index; CHAOS, Congenital High Airway Obstruction Syndrome; PPROM, preterm premature rupture of membranes.

In all cases, diagnostic characterization was performed using both two‐ and three‐dimensional ultrasound and fetal MRI. Teratomas typically appeared as large (median tumor size of 5.8 cm, IQR 3.0, 8.0) complexes with mixed solid and cystic components, often with irregular septations and echogenic foci suggestive of calcification, two of which corresponded to epignathus arising in the oropharyngeal–submandibular region and the remaining case was an anterolateral cervical thyroid teratoma. They showed significant distortion or compression of upper airway structures, including tracheal deviation, mandibular displacement, and oropharyngeal obstruction. One fetus presented with associated fetal hydrothorax, pericardial effusion, and cardiomegaly. Lymphatic malformations are characterized by multiloculated, predominantly cystic lesions with thin or irregular septa, often involving multiple cervical compartments. Most were large (median tumor size of 10.0 cm, IQR 7.2, 12.5), left‐sided or midline, and in some cases extended into the intrathoracic regions. Variable degrees of vascularity were noted, with some lesions demonstrating a feeding vessel arising from the carotid artery. The cervical bronchogenic cyst presented as a well‐defined anechoic lesion of 5.2 × 2.8 cm, located in the anterior‐upper mediastinal cervical region, with internal incomplete septation. Ultrasound and MRI revealed tracheal displacement and polyhydramnios, raising concerns for potential airway obstruction despite the suspected esophageal or bronchogenic origin of the lesion. Histological analysis postnatally confirmed the diagnosis of bronchogenic cysts with areas of squamous metaplasia. In the fetus diagnosed with CHAOS secondary to laryngeal atresia, ultrasound revealed typical features including bilateral lung hyperexpansion, tracheobronchial dilation, diaphragmatic inversion, preserved vocal cord mobility and fluid accumulation in the oropharynx. MRI confirmed complete upper airway obstruction and suggested tracheal hypoplasia. Finally, in the case of the Pierre Robin sequence, prenatal imaging showed marked retrognathia with mandibular hypoplasia, glossoptosis, and palatal clefting. Quantitative measurements such as a facial angle of 38° (below −2 SD) and a mandibular index of 26.5% supported the diagnosis.

The procedure was performed in an elective fashion in all cases. Technical data on the procedures are shown in Table 2. Most patients were managed with combined general and peridural anesthesia. In one case ‐diagnosed with bronchogenic cyst‐fetoscopic evaluation confirmed patency of the airways after intra‐procedural cystic puncture. Following multidisciplinary team discussion, a standard cesarean section was considered appropriate given the anticipated low risk of airway compromise and the planned delivery in a controlled setting with immediate access to specialized neonatal airway management. The infant exhibited no signs of respiratory distress postnatally. In the other 8 cases, EXIT was decided after fetoscopy. Of these, in seven cases (77.8%), visual inspection confirmed a trajectory to reach the larynx through orotracheal intubation. In two cases, fetoscopic access to the larynx proved particularly challenging. To facilitate subsequent airway management, a catheter was advanced into the trachea through the working channel of the fetoscope. The fetoscope was then carefully removed while ensuring that the catheter remained in position, providing a guide for tracheal intubation during the EXIT procedure. Orotracheal intubation was subsequently performed successfully during EXIT in all seven cases. Finally, in the case diagnosed with CHAOS, laryngeal dysplasia was confirmed by direct visualization, so that intra‐EXIT tracheostomy was performed. Fetal monitoring during EXIT was performed by combining ultrasonography and pulse oximetry (4) or ultrasound alone (4). During EXIT, only one fetus presented bradycardia at the end of the procedure but recovered after successful intubation. Median birth weight was 2775 g (IQR 2,300, 3000), and median umbilical artery pH was 7.16 (IQR 7.10, 7.22). One mother presented postpartum hemorrhage, requiring additional uterotonic management and red blood cell transfusion, without further consequences.

**TABLE 2:** Characteristics of the fetoscopic‐EXIT procedures.

	*n* = 9
Gestational age at delivery (weeks + days)	35^+6^ (34^+4^, 37^+1^)
Maternal anesthesia	
General & peridural	6 (66.7%)
General	2 (22.2%)
Peridural	1 (11.1%)
Fetal airway modalities	
Orotracheal intubation	7 (77.8%)
Tracheostomy	1 (11.1%)
Spontaneous	1 (11.1%)
Fetal monitorization during EXIT	
Pulse‐oximeter & ultrasound	4 (44.4%)
Only ultrasound	4 (44.4%)
None	1 (11.1%)
Fetal instability during EXIT	1 (11.1%)
EXIT duration (minutes)	17 (8, 25)
Fetoscopy duration (minutes)	14 (3, 23)
Intubation duration (minutes)	8 (1, 17)

*Note:* Categorical variables are described as number (%), and numerical variables as median (Q1, Q3).

Abbreviation: EXIT, Ex‐Utero Intrapartum Treatment.

Regarding neonatal evolution, there was one neonatal death 3 hours after birth due to severe pulmonary insufficiency with impossibility to achieve normal ventilatory parameters. On necropsy, the newborn was diagnosed with severe pulmonary hypoplasia, likely attributed to diaphragmatic paralysis resulting from chronic compression of the phrenic nerves at the neck. The remaining 8 newborns showed normal respiratory function under intubation, allowing surgical intervention of the cervical masses where appropriate. Surgical treatments could be performed in one operation in some cases, while in others required two or more procedures. The newborn with Pierre‐Robin syndrome eventually underwent tracheostomy on the decision of the managing physicians. Laryngeal dysplasia was managed by several interventions and a laryngeal stent at 2 years. At a median follow‐up of 6 years (IQR 3–7 years), all children demonstrated normal growth and neurological development according to pediatric reports. Therefore, intact survival was achieved in 88.8% of cases in this series.

## 5 Discussion

This study describes the role of pre‐EXIT fetoscopic airway evaluation in fetuses with suspected airway obstruction detected during gestation. Fetoscopy is a feasible technique that can function as a decision‐shaping adjunct in selected cases, enabling de‐escalation of management when airway patency is confirmed, thereby avoiding EXIT, and optimizing airway planning by delineating the route of orotracheal intubation, eventually avoiding tracheostomy. Importantly, no significant maternal complications were observed, and perinatal outcomes were favorable in most cases described.

Consistent with prior literature, the most frequent lesions in our cohort were cervical teratomas and lymphatic malformations. This study highlights the diverse anatomical causes of fetal airway obstruction, ranging from extrinsic tumors to intrinsic defects and craniofacial abnormalities, and highlights the utility of fetoscopy across this spectrum. Notably, severe micrognathia has increasingly been recognized as a potential indication for EXIT when associated with prenatal findings suggestive of upper airway obstruction, such as polyhydramnios or imaging evidence of posterior pharyngeal narrowing or collapse. In our series, this scenario was represented by a fetus with the Pierre Robin sequence and prenatal evidence of airway compromise. Porter et al. identified severe micrognathia as the most frequent indication for EXIT in their cohort [2]. However, not all fetuses with micrognathia or the Pierre Robin sequence require EXIT, and management should be individualized according to the predicted risk of airway obstruction. Our findings suggest that fetoscopy may be a useful adjunct in selected high‐risk cases.

Prior research has established that fetoscopy is not only useful for airway assessment but can also be therapeutic. It was first described by Kohl for tracheal decompression in CHAOS associated with Fraser syndrome [14]. Our group was the first to report its successful use in bronchial atresia producing severe thoracic compression with mediastinal shift, pulmonary hypoplasia, and fetal hydrops [15]. In that case, fetal tracheoscopy enabled access to the right main bronchus, which was perforated with a laser to create communication with the intrapulmonary bronchus; this led to a marked reduction in the thoracic mass effect and resolution of hydrops. Additionally, we have also applied a fetoscopic approach in CHAOS, assessing the larynx and vocal cords to differentiate glottic from subglottic obstruction, and in cases of isolated tracheal web using fetoscopy to perforate it, resulting in normalization of lung size and resolution of hydrops [16]. In the current results, we show a case with a cervical cystic lesion that was percutaneously punctured prior to fetoscopy to achieve decompression, after which fetoscopy is used to confirm airway patency, thereby obviating airway securing during delivery.

Additionally, fetoscopy may enable in‐utero airway restoration. Cruz et al. described FETI (Fetal Endoscopic Tracheal Intubation), in which a semi‐rigid fetoscope is advanced trans‐orally into the trachea to place an endotracheal tube, allowing conventional cesarean with cannula position maintained for immediate postnatal ventilation, thus avoiding EXIT and reducing operative time, bleeding, and EXIT‐related risks [13]. Our experience also supports that fetoscopic airway evaluation refines the airway management strategy by enabling, in selected scenarios, the placement of a catheter/guide to facilitate intubation and by informing the need for direct tracheostomy or bronchoscopy in suspected difficult airways.

Appropriate prenatal assessment of fetuses at risk of airway obstruction requires individualized, multidisciplinary planning to optimize neonatal outcomes. When imaging suggests uncertain airway patency, fetoscopy can guide the decision to proceed with EXIT versus conventional delivery, as demonstrated by Beckers et al. among 11 cases, eight underwent prior fetoscopy and in six airway patency was confirmed, so only two required EXIT [17]. Although in our cohort only one EXIT was ultimately avoided after fetoscopic evaluation, this likely reflects strict preselection for EXIT based on ultrasound and MRI.

While fetoscopy has traditionally been emphasized in the setting of large head and neck masses, we found it equally valuable when facial anatomy is altered or when complete airway obstruction is suspected. Collectively, our data aligns with prior cohorts and adds to the growing evidence that fetoscopy should be integrated as both a diagnostic and, in selected cases, a therapeutic modality, helping to refine indications for EXIT. Given that EXIT, though lifesaving, carries maternal and fetal risks, reserving it for confirmed or highly probable obstruction is essential.

This is a small, heterogeneous series and may not reflect clinically meaningful differences among the various indications for EXIT, limiting generalizability. Ideally, multicenter cohorts evaluating comparable patients with similar pathological presentations are needed to clarify which fetuses benefit the most from fetoscopic airway evaluation.

## 6 Conclusion

Fetoscopic airway evaluation is a valuable tool in the context of EXIT management of fetuses with potential airway obstruction. Beyond diagnostic confirmation, its use can directly influence therapeutic strategy, improve airway access, and in selected cases, eliminate the need for EXIT. Integration of fetoscopy into EXIT protocols offers a more individualized and safer approach in high‐risk airway cases when performed in experienced fetal surgery centers.

## Funding

The research leading to these results has received funding from Instituto de Salud Carlos III (ISCIII) through the project “FI23/00159”, and co‐funded by the European Union.

## Ethics Statement

The study was approved by the Institutional Ethics Committee (approval code: HCB/2025/1083) on October 23, 2025. All procedures were conducted in accordance with the ethical standards of the institutional research committee and with the principles of the Declaration of Helsinki.

## Consent

Patient consent statement is not applicable or needed for this study. Due to the retrospective and observational nature of the study, the requirement for written informed consent was waived by the Ethics Committee.

## Conflicts of Interest

The authors declare no conflicts of interest.

## Data Availability

The data that support the findings of this study are available from the corresponding author upon reasonable request.
